# The Relationship Between Self-Esteem and Burnout Among Operating Room Nurses

**DOI:** 10.3390/nursrep16060193

**Published:** 2026-06-05

**Authors:** Viktorija Zalpyte, Indre Cergelyte-Podgrusiene

**Affiliations:** Department of Nursing and Midwifery, Institute of Health Sciences, Faculty of Medicine, Vilnius University, 01513 Vilniaus, Lithuania; indre.cergelyte-podgrusiene@mf.vu.lt

**Keywords:** operating room nurses, perioperative nurse, scrub nurse, nurse anesthetist, burnout, moral exhaustion, self-esteem, self-worth

## Abstract

**Background:** Operating room nurses are exposed to high levels of occupational stress, making them particularly vulnerable to burnout. Psychological resources such as self-esteem may play a protective role; however, evidence in perioperative settings remains limited. **Objective:** This study aimed to examine the relationship between self-esteem and burnout among operating room nurses. **Methods:** A quantitative cross-sectional study was conducted among 261 operating room nurses working in public healthcare institutions in Vilnius, Lithuania. Self-esteem was measured using the Rosenberg Self-Esteem Scale (RSES), and burnout was assessed using the Burnout Assessment Tool (BAT-23). Data were analyzed using non-parametric tests, Spearman’s correlation, hierarchical regression analysis, and Bonferroni correction for multiple comparisons. **Results:** A statistically significant negative association was found between self-esteem and burnout (*p* < 0.001). Higher self-esteem was associated with lower levels of exhaustion (r = −0.326), emotional distress (r = −0.357), cognitive impairment (r = −0.398), and psychological disengagement (r = −0.331). The strongest associations were observed for secondary symptoms (r = −0.420) and overall burnout (r = −0.410). In regression analysis, self-esteem remained a significant predictor of burnout (β = −0.438, *p* < 0.001), explaining a substantial proportion of variance. **Conclusions:** Self-esteem is a significant protective factor against burnout among operating room nurses. Interventions aimed at strengthening psychological resources may contribute to reducing burnout in high-intensity clinical environments.

## 1. Introduction

Professional burnout syndrome (PBS) among healthcare workers is considered a global problem—data from the literature shows that the prevalence of burnout in the nursing community ranges from 33% to 79% on average, depending on the country and the prevailing working conditions of nurses [1,2,3]. According to the World Health Organization, burnout is a syndrome resulting from chronic workplace stress that has not been successfully managed. It is characterized by feelings of energy depletion or exhaustion, increased mental distance from one’s work, and reduced professional efficacy. Burnout develops gradually and may negatively affect an individual’s psychological well-being, work performance, and overall quality of life [4].

The professional practice of operating room nurses (ORNs) is characterized by specific job demands and constant vigilance. However, the principle of teamwork does not always lead to exclusively positive outcomes: conflicts may arise among team members, disagreements may occur regarding role responsibilities, and unresolved organizational issues increase job dissatisfaction and even the intention to leave the profession [5]. These factors are associated with an increased risk of burnout, as they directly negatively affect the psychological well-being of ORNs.

The operating room consists of surgical and anesthesiology teams; therefore, it includes nurses of different specialties, such as scrub nurses (SNs) and nurse anesthetists (NAs). Although these professionals work closely together, their roles differ. SNs are directly involved in surgical procedures, ensuring the preparation and proper use of instruments during surgery [6], whereas NAs are responsible for continuous monitoring of the patient’s condition and ensuring the safe administration of anesthesia throughout the perioperative period [7]. Thus, it can be argued that these significant occupational differences may lead to variations in stress levels and have an unequal impact on the development of PBS. Based on a literature review of studies published between 2013 and 2023, the estimated prevalence of PBS NAs ranges from 12.5% to 72%. The most important risk factors are related to the work environment, including lack of autonomy and leadership, as well as poor interpersonal relationships within the team [8]. Other studies confirm the importance of work-related stress in the development of PBS. It has been found that a large proportion of ORNs (70.3%) experience high levels of stress, which are associated with an increased risk of burnout [9].

In Lithuania, most nurses are employed full-time, typically working 38.5 h per week. Hospital nursing schedules commonly involve rotating shifts, including 6–8 h shifts, 12 h day or night shifts, and, in some settings, 24 h shifts [10]. Operating room nurses work in a demanding environment that requires sustained concentration, rapid decision-making, and close collaboration within multidisciplinary surgical teams. Such working conditions may contribute to physical and psychological strain. Previous research conducted among healthcare professionals in Lithuania has shown that a higher number of working hours per week is significantly associated with more severe burnout syndrome [11]. Therefore, workload and working time may be important factors contributing to burnout among operating room nurses.

Burnout prevalence among nurses is relatively similar across the Baltic countries. Data from a study conducted in Latvia revealed that nearly half of the participating nurses experienced moderate levels of burnout (49.5%), with the greatest influence attributed to exhaustion and work environment factors, such as disorganized teamwork, excessive workload, and lack of leadership [3]. Studies conducted in Lithuania indicate that burnout among nurses is predominantly characterized by moderate levels, with the most prominent features being exhaustion related to physical fatigue, emotional irritability, and cognitive impairment [12]. More recent studies in the Baltic region continue to demonstrate a high prevalence of burnout among nurses. Based on data from a study conducted in Lithuania in 2023, approximately 74% of nurses were found to exhibit symptoms characteristic of burnout [11]. Furthermore, although burnout levels among healthcare professionals remain consistently high, occupational stress levels are rapidly increasing. Recent evidence from Lithuania and Poland suggests that the ongoing geopolitical situation related to the war in Ukraine may contribute to increased work-related stress among nurses [13]. Increased occupational stress may, in turn, represent an additional factor associated with burnout.

However, even with the elimination of the aforementioned risk factors, it remains essential to strengthen nurses’ internal psychological resources and to focus on protective factors that mitigate burnout. Self-esteem refers to an individual’s overall evaluation of their own worth and reflects attitudes and beliefs about oneself. Higher self-esteem has been associated with greater psychological resilience, more effective coping with stress, and better mental well-being [14]. Studies indicate that higher self-esteem enhances self-confidence among both practicing nurses and nursing students, strengthening their ability to cope with stressful situations, and thereby significantly contributing to reducing burnout and promoting psychological well-being [15,16]. Accordingly, this study aimed to explore the relationship between self-esteem and burnout among operating room nurses. It was hypothesized that self-esteem and sociodemographic characteristics would be significantly associated with burnout among operating room nurses.

## 2. Materials and Methods

### 2.1. Research Design and Participants

A quantitative cross-sectional study was conducted. Data were collected between November 2025 and February 2026 in public healthcare institutions in Vilnius. The study population consisted of SNs and NAs (*N* = 727). The population size was determined using workforce data obtained from the annual reports of the participating healthcare institutions and information provided by their human resources departments. Participants were selected using convenience sampling. The sample size was calculated using Paniotto’s formula for a finite population (*n* = *N*/(1 + *N* × Δ^2^), where *n* is the required sample size, *N* is the population size, and Δ is the margin of error. Assuming a population size of 727 scrub nurses and nurse anesthetists and a margin of error of 5% (Δ = 0.05), the minimum required sample size was 258 respondents.

Nurses included in the study were: (1) registered nurses qualified as scrub nurses; (2) registered nurses qualified as nurse anesthetists; and (3) operating room nurses able to understand Lithuanian.

Nurses excluded from the study were: (1) scrub nurses and nurse anesthetists not employed in public healthcare institutions in Vilnius, and (2) nurses who were on annual leave, sick leave, or parental leave during the study period.

### 2.2. Data Collection

A mixed-method data collection approach was used. Participants were recruited through the Lithuanian Association of Operating Room Nurses and the Lithuanian Association of Anaesthesia and Intensive Care Nurses, which are the principal professional associations representing nurses working in perioperative, anesthesia, and intensive care settings in Lithuania. Dissemination of the electronic questionnaire through these organizations enabled access to the target population and facilitated recruitment of nurses with relevant professional experience. In addition, paper questionnaires were distributed directly in healthcare institutions employing operating room nurses.

A total of 163 paper questionnaires were distributed in participating departments, of which 135 were returned. Twenty-eight questionnaires were returned blank, and four were excluded due to incomplete responses and missing data. In addition, 158 participants completed the questionnaire electronically. The final analytical sample consisted of 261 operating room nurses (*n* = 261). The response rate for paper questionnaires was 82.8%; the response rate for the electronic survey could not be calculated because the exact number of nurses who received the invitation was unknown.

### 2.3. Measures

The questionnaire consisted of 50 items organized into three sections. The first section included seven questions on respondents’ sociodemographic and professional characteristics, including sex, age, education, work experience, position in the operating room, surgical specialty, and workload. The second section comprised the Rosenberg Self-Esteem Scale (RSES), developed by M. Rosenberg (1965) [17], consisting of 10 items assessing global self-esteem. Responses were rated on a 4-point Likert scale ranging from 0 (“strongly disagree”) to 3 (“strongly agree”), with total scores ranging from 0 to 30; higher scores indicated higher self-esteem. The third section included the Burnout Assessment Tool (BAT), developed by Schaufeli et al. [18], consisting of 33 items evaluating burnout symptoms across the dimensions of exhaustion, mental distance, cognitive impairment, emotional impairment, and secondary symptoms, including psychological distress and psychosomatic complaints. BAT items were rated on a 5-point Likert scale ranging from 0 (“never”) to 4 (“always”), with higher scores indicating greater burnout symptom severity. The Lithuanian version of the BAT (BAT-LT) has demonstrated satisfactory reliability and validity in a Lithuanian working population [19].

Both the RSES and BAT are internationally validated instruments with established psychometric properties and have been widely used in scientific research. The internal consistency of the instruments in the present study was assessed using Cronbach’s alpha coefficient. The RSES demonstrated good internal consistency (α = 0.863). For the BAT, very good internal consistency was observed for the exhaustion (α = 0.91) and cognitive impairment (α = 0.91) subscales, while good internal consistency was found for the mental distance (α = 0.81), emotional impairment (α = 0.86), and secondary symptoms (α = 0.87) subscales. The overall BAT scale demonstrated excellent internal consistency (α = 0.94).

### 2.4. Statistical Analysis

Data analysis was performed using IBM SPSS Statistics version 31.0.2.0 (IBM Corp., Armonk, NY, USA). Normality was assessed using the Shapiro–Wilk test. As the data did not meet the assumptions of normality, non-parametric tests were applied. Descriptive statistics were calculated. Differences between scrub nurses and nurse anesthetists were assessed using the Mann–Whitney U test. Spearman’s correlation coefficient was used to assess relationships between self-esteem and burnout dimensions. Because multiple correlations were performed, Bonferroni correction was considered, and the adjusted significance threshold for correlational analyses was set at *p* < 0.008. Prior to hierarchical regression analysis, the assumptions of linearity, normality of residuals, homoscedasticity, independence of errors, and absence of multicollinearity were assessed. Visual inspection of residual plots and diagnostic statistics indicated that all assumptions were satisfied. Hierarchical regression analysis was conducted to evaluate the association between self-esteem and burnout. Statistical significance was set at *p* < 0.05.

### 2.5. Ethical Considerations

Participation in the study was voluntary and anonymous. Before completing the questionnaire, participants received information about the study objectives, procedures, confidentiality, and the voluntary nature of participation, including their right to discontinue participation at any time without consequences. Completion of the questionnaire was considered an indication of an individual’s informed consent to participate in the study. The study was approved by the Biomedical Research Ethics Committee of the Faculty of Medicine, Vilnius University (No. (1.7 E) 150000-KT-565, 16 October 2025). The study was conducted in accordance with the ethical principles of the Declaration of Helsinki (1975, revised in 2008) and the General Data Protection Regulation (GDPR, Regulation (EU) 2016/679).

## 3. Results

A total of 261 complete questionnaires were included in the analysis. The majority of participants were female (95.8%). The largest age group was 46–55 years (30.7%), while those aged 26–35 and ≥56 each accounted for 21.1%. Most participants worked as scrub nurses (60.9%), with the remainder employed as nurse anesthetists (39.1%). Over half of the participants held a bachelor’s degree (54.4%) (Table 1).

### 3.1. Assessment of Self-Esteem Among Operating Room Nurses

Self-esteem scores by specialization are presented in Table 2. The median self-esteem score was identical in both groups: 23 (19–26) among scrub nurses and 23 (20–26) among nurse anesthetists. Mean values were also comparable. A Mann–Whitney U test confirmed that there was no statistically significant difference in self-esteem scores between scrub nurses and nurse anesthetists (U = 7512.5, Z = −1.005, *p* = 0.315).

Most participants demonstrated a moderate level of self-esteem (SNs = 69.2%, NAs = 70.6%). Low self-esteem was observed in a small proportion of participants (SNs = 4.4%, NAs = 3.9%), while high self-esteem was reported by approximately one-quarter of respondents (SNs = 26.4%, NAs = 25.5%).

### 3.2. Assessment of Burnout Among Operating Room Nurses

Burnout characteristics by specialization are presented in Table 3. Most participants in both groups demonstrated a moderate level of burnout (SNs = 55.3%, NAs = 53.9%).

Low burnout levels were more common among nurse anesthetists (24.5%) compared to scrub nurses (6.9%), whereas high burnout levels were more prevalent among scrub nurses (31.4%) than nurse anesthetists (18.6%). Very high burnout was observed in a small proportion of participants in both groups (SNs = 6.3%, NAs = 2.9%). A Mann–Whitney U test revealed a statistically significant difference in burnout scores between SNs and NAs (U = 6029.5, Z = −3.496, *p* < 0.001), indicating higher burnout levels among SNs.

### 3.3. Association Between Nurses’ Self-Esteem and Burnout

A statistically significant negative correlation was observed between the self-esteem of ORNs and all burnout dimensions (*p* < 0.001) (Table 4). Higher self-esteem was associated with lower levels of emotional distress (r = −0.357), cognitive impairment (r = −0.398), mental distance (r = −0.331), and exhaustion (r = −0.326). The strongest associations were found for secondary symptoms (r = −0.420) and total burnout score (r = −0.410).

Because multiple correlations were performed, a Bonferroni correction was considered. The adjusted significance threshold for the correlational analyses was set at *p* < 0.008. All reported correlations remained statistically significant after correction.

Prior to hierarchical regression analysis, all assumptions were examined and met. Residuals were normally distributed, homoscedasticity and linearity assumptions were satisfied, independence of errors was confirmed (Durbin-Watson = 1.873), and no multicollinearity was detected (VIF = 1.008 − 1.054).

Hierarchical regression analysis was performed to determine the prevalence of burnout predictors in the ORNs (Table 5). In the first model, sociodemographic variables (specialization, age, gender and education) explained 12.6% of the burnout variation (R^2^ = 0.126, *p* < 0.001).

The second model, with self-esteem of ORNs included, explained how variations in burnout in the sample increased to 30.9% (R^2^ = 0.309; ΔR^2^ = 0.183; *p* < 0.001). Thus, we see that self-esteem is a significant negative factor in the prognosis of nurse burnout (β = −0.438, *p* < 0.001), indicating that an increase in self-esteem reduces the burnout score of nurses.

It was also found that when self-esteem was included in the regression model, age (*p* < 0.001) and nurse specialization (*p* = 0.001) remained significant from sociodemographic variables, while gender and education were not statistically significant.

## 4. Discussion

The aim of this study was to assess the relationship between self-esteem and burnout among nurses working in an operating room. The results showed that higher self-esteem is associated with lower levels of burnout. The findings contribute to existing evidence demonstrating that positive self-esteem improves emotional well-being and reduces psychological distress in nurses [14,15].

The strongest associations were found between self-esteem and secondary burnout symptoms, including psychological distress and psychosomatic complaints, as well as the total burnout score. Similar results were reported by Duran et al. (2021), who found that higher self-esteem is associated with lower psychological distress [20]. This supports the view that self-esteem may act as a protective factor against burnout not only at the emotional but also at the somatic level. The findings of this study indicate that higher self-esteem among nurses is significantly associated with lower levels of burnout, independent of sociodemographic factors. This is consistent with previous research suggesting that psychological resources such as self-esteem may play a protective role in the development of burnout [13,16]. In addition, nurses with higher self-esteem are more likely to demonstrate effective coping strategies, greater emotional resilience, and a more positive self-perception, which may help them manage work-related stress more effectively [17].

The results also showed differences between groups of operating room nurses: scrub nurses demonstrated higher levels of burnout, whereas nurse anesthetists reported lower levels. These differences may be explained by variations in job roles, responsibilities, and work environment factors such as teamwork, fatigue, anxiety, and organizational challenges [2,18]. In addition, differences in physical workload may have contributed to the observed results. Scrub nurses are often required to stand for prolonged periods during surgical procedures, maintain sterile conditions, handle surgical instruments, and continuously respond to the needs of the surgical team [10]. These responsibilities may impose greater physical demands compared to those experienced by nurse anesthetists and could partly explain the differences in burnout levels observed between the two groups. Previous studies have shown that prolonged standing, physically demanding tasks, and awkward working postures may contribute to fatigue, musculoskeletal problems, and increased work-related stress, which are recognized risk factors for burnout among operating room personnel [21].

The study findings suggest that strengthening psychological resources and resilience may help reduce burnout risk, particularly in high-intensity clinical environments. Future research should explore self-esteem and burnout relationships using longitudinal designs and include additional variables such as teamwork, organizational environment, and social support.

The study has several limitations. Self-esteem and burnout were assessed at the same time, so causal relationships or their direction between the variables cannot be established. In addition, the data were collected using self-rating scales, which are likely to be subject to response bias. Finally, the study was conducted only among operating room nurses, so the results may have limited applicability to other nursing communities.

## 5. Conclusions

Self-esteem in operating room nurses is associated with lower burnout, especially when assessing psychological distress and psychosomatic complaints. Scrub nurses were also found to have higher burnout rates compared to nurse anesthetists. Such results suggest that strengthening self-esteem among operating room personnel can have a positive impact on reducing burnout.

## Figures and Tables

**Table 1:** Sociodemographic characteristics of participants (*N* = 261).

Variable	Category	n (%)
Gender	Male	11 (4.2)
	Female	250 (95.8)
	Non-binary/third gender	0
	Prefer not to say	0
Age	≤25	24 (9.2)
	26–35	55 (21.1)
	36–45	47 (18)
	46–55	80 (30.7)
	≥56	55 (21.1)
Specialization	Nurse anesthetist	102 (39.1)
	Scrub nurse	159 (60.9)
Education	Professional bachelor’s degree	142 (54.4)
	University bachelor’s degree	79 (30.3)
	Master’s degree	40 (15.3)

**Table 2:** Self-esteem characteristics of operating room nurses.

Characteristic	Scrub Nurses (SNs) (n = 159)	Nurse Anesthetists (NAs) (n = 102)
Minimum score	8	11
Maximum score	30	30
Median (Q1–Q3)	23 (19–26)	23 (20–26)
Mean (SD)	22.27 (4.53)	22.91 (4.29)
Low self-esteem (%)	4.4	3.9
Moderate self-esteem (%)	69.2	70.6
High self-esteem (%)	26.4	25.5

**Table 3:** Burnout characteristics among operating room nurses.

Characteristic	Scrub Nurses (SNs) (*n* = 159)	Nurse Anesthetists (NAs) (*n* = 102)
Minimum score	1.04	1.00
Maximum score	3.83	3.70
Median (Q1–Q3)	2.22 (1.83–2.61)	1.96 (1.60–1.90)
Mean (SD)	2.26 (0.58)	2.02 (0.56)
Low burnout (%)	6.9	24.5
Moderate burnout (%)	55.3	53.9
High burnout (%)	31.4	18.6
Very high burnout (%)	6.3	2.9

**Table 4:** Correlations between self-esteem and burnout.

Variable	Spearman’s r	*p*
Self-esteem and exhaustion	−0.326	<0.001 *
Self-esteem and emotional distress	−0.357	<0.001 *
Self-esteem and cognitive impairment	−0.398	<0.001 *
Self-esteem and mental distance	−0.331	<0.001 *
Self-esteem and secondary symptoms	−0.420	<0.001 *
Self-esteem and total burnout score	−0.410	<0.001 *

* Statistically significant after Bonferroni correction for multiple comparisons (adjusted *p* < 0.008).

**Table 5:** Factors predicting burnout of nurses working in the operating room: analysis of hierarchical regression.

Variable	B	SE	β	*p*
Model 1				
Gender	−0.412	0.170	−0.143	0.016 *
Age	−0.116	0.027	−0.255	<0.001 *
Specialization	−0.235	0.070	−0.198	<0.001 *
Education	0.023	0.033	0.041	0.492
Model 2				
Gender	−0.241	0.153	−0.083	0.116
Age	−0.102	0.024	−0.223	<0.001 *
Specialization	−0.202	0.062	−0.170	0.001 *
Education	0.058	0.030	0.105	0.051
Self-esteem	−0.057	0.007	−0.438	<0.001 *

* Statistically significant (*p* < 0.05); Model 1: R^2^ = 0.126, adjusted R^2^ = 0.112, F = 9.210, *p* < 0.001; Model 2: R^2^ = 0.309, adjusted R^2^ = 0.295, ΔR^2^ = 0.183, F = 22.776, *p* < 0.001.

## Data Availability

The data presented in this study are available on request from the corresponding author.

## Public Involvement Statement

There was no public involvement in any aspect of this research.

## Guidelines and Standards Statement

This manuscript was prepared in accordance with the STROBE (Strengthening the Reporting of Observational Studies in Epidemiology) guidelines for cross-sectional studies [22].
